# Leukocytes and systemic inflammatory response syndrome as prognostic factors in pulmonary embolism patients

**DOI:** 10.1186/1471-2466-13-74

**Published:** 2013-12-10

**Authors:** Jun Yeon Jo, Mi Young Lee, Jin Wook Lee, Byung Hak Rho, Won-Il Choi

**Affiliations:** 1 Pulmonary Unit, Department of Internal Medicine, Dongsan Hospital, Keimyung University School of Medicine, Daegu, Korea; 2 Department of Preventive Medicine, Keimyung University School of Medicine, Daegu, Korea; 3 Department of Radiology, Dongsan Hospital, Keimyung University School of Medicine, Daegu, Korea

**Keywords:** Leukocytosis, Pulmonary embolism, Systemic inflammatory response syndrome

## Abstract

**Background:**

Hemodynamic status and cardiac function are important factors for predicting pulmonary embolism (PE) prognosis. Although inflammation is considered a risk factor for deep vein thrombosis, the prognostic significance of both systemic inflammatory response syndrome (SIRS) and leukocytosis has not been elucidated. This study evaluates PE prognostic factors, including SIRS and leukocytes.

**Methods:**

This retrospective cohort study included 667 PE patients. Risk evaluation included SIRS and leukocytosis. A prediction model was developed based on independent predictors of 30-day mortality.

**Results:**

Fifty-seven patients (8.5%) died within 30 days of PE. Multivariate analysis showed that SIRS satisfying the WBC criteria (odds ratio [OR], 2.8; 95% confidence interval [CI], 1.5–5.4), altered mental status (OR, 4.0; 95% CI, 1.8–8.7), shock (OR, 2.6; 95% CI, 1.0-7.1), and right-to-left ventricle diameter ratio (OR, 1.7; 95% CI, 1.0-2.8) were associated with 30-day mortality. SIRS criteria, including body temperature (OR, 4.6; 95% CI, 1.4–14.8), heart rate (OR, 2.0; 95% CI, 1.1–3.6), respiratory rate (OR, 2.5; 95% CI, 1.4–4.6), and white blood cells (WBC) count (OR, 1.9; 95% CI, 1.2–3.5) predicted short-term mortality following PE. The area under the receiver operating characteristic curve for the prognostic model performance was 0.76 (95% CI, 0.66–0.85); pulmonary embolism severity index (PESI) and PESI + WBC count were 0.72 (95% CI, 0.68–0.75) and 0.76 (95% CI, 0.72–0.79, *P* < 0.001 versus PESI), respectively.

**Conclusions:**

Leukocytosis and SIRS are important factors in determining short-term outcomes in PE patients.

## Background

Hemodynamic status and comorbidities are key factors in the prognosis of pulmonary embolism (PE) [1,2]. In addition to hemodynamic variables, cardiac biomarkers such as troponins and natriuretic peptides are risk factors for patients with acute PE [3]. The PE prognostic prediction model that is based on these variables is widely accepted [2,4].

Initial risk stratification of patients with PE is based on the presence of shock or hypotension [5]. If the patient is hemodynamically stable, right ventricular function is then assessed by echocardiography, and cardiac biomarkers are measured [5]. However, these variables are not accurate predictors of PE mortality, especially in hemodynamically stable patients [6,7].

The triad of vessel wall injury, venous stasis, and blood hypercoagulability has historically been considered a major risk factor for venous thrombosis [8]. Infection is another established risk factor for PE [9], and in certain cases, PE has been associated with influenza [10] or cytomegalovirus infection [11].

In deep vein thrombosis (DVT), an inflammatory reaction triggers endothelial cell dysfunction [12,13] and results in high serum concentrations of the inflammatory marker C-reactive protein [14]. Such inflammatory reactions frequently induce well-studied DVT risk factors; however, few studies have investigated a prognostic prediction model for PE. Respiratory and pulse rates are included in the representative PE severity index (PESI) [15]. Nevertheless, there is a dearth of studies on the potential association between the systemic inflammatory response and PE patient prognosis.

Studies have suggested that leukocytes contribute to venous thrombosis by damaging the endothelium [16,17]. Animal models have shown that genetic knock-out of the adhesion molecules E- and P-selectin results in a reduction in thrombus size, which is associated with altered leukocyte accumulation in the surrounding vein wall [18]. However, the role of leukocytes in the prognosis of PE has not been well studied. We hypothesize that both a systemic inflammatory response and leukocytosis may be negative prognostic factors in PE patients.

## Methods

### Study design

This retrospective single center observational cohort study was conducted between January 2005 and December 2011. This study was approved by the institutional review board at Dongsan Hospital, Keimyung University School of Medicine.

### Study subjects

A total of 667 PE patients were enrolled at Dongsan Hospital from January 2005 through December 2011. All PE patients treated during the study period were included; no additional selection criteria were used. Subjects were either admitted to the hospital or were Emergency Department or outpatient clinic patients.

### Image studies

PE was defined either as a filling defect in the pulmonary artery detected through chest computed tomography (CT) or CT pulmonary angiography, or diagnosed based on a ventilation perfusion scan. DVT diagnosis was confirmed via ultrasound examination of the lower extremity veins in patients with clinically suspected PE.

### Study methods

Electronic medical records of all PE patients were examined. Risk factors included renal dysfunction, defined as serum creatinine level >1.3 mg/dL; active cancer, defined as treatment with an anti-cancer agent within 3 months of PE diagnosis; hospital admission for supportive therapy within 3 months of PE diagnosis; or outpatient use of analgesics for end-stage malignancy. Shock was defined as systolic blood pressure of <90 mmHg.

CT was performed using a 16 or 64-slice detector. The reconstruction interval of the scan was 3 mm. The ratio of right ventricular diameter to left ventricular diameter (RV/LV ratio) was calculated using CT scan images showing the interventricular septum and myocardium from the longitudinal axis of the heart [19,20]. A radiologist reviewed all the CT scans in a blind fashion.

Patients were diagnosed with systemic inflammatory response syndrome (SIRS) if they met 2 or more of the following criteria: peripheral white blood cell (WBC) count of <4,000/μL or >12,000/μL, respiratory rate >20 breaths per min, pulse rate >90 beats per min, and body temperature of >38.3°C or <36.0°C [21]. We retrospectively collected the clinical characteristics and laboratory data on factors such as comorbidity, symptoms, chest CT findings, vital signs, RV/LV ratio, NT Pro-BNP, and complete blood count at the time of diagnosis of PE.

We defined 30-day all-cause mortality as a primary outcome.

### Statistical analysis

All values are expressed as mean ± standard deviation. Data were analyzed using SAS version 9 (SAS Institute, Cary, NC, USA) and MedCalc version 11.0 (MedCalc Software, USA). A χ^2^ test was used to compare frequencies. Student’s *t* test was used for statistical significance analysis of continuous variables, and cross-analysis was used for categorical variables. Statistical significance was set at <0.05. Univariate analysis of statistically significant variables was conducted using logistic regression. Multivariate analysis was performed for variables with a *p-*value of <0.1. We used the area under the receiver operating characteristic (ROC) curve or the area under the curve (AUC) to quantify the ability of the model to distinguish between high- and low-risk subjects. To compare the ROC curves, we used the Delong et al. [22] method to calculate the standard error of the AUC and the difference between 2 AUCs with the exact binomial confidence interval for the AUCs. The improved discriminative and predictive values of the WBC count + PESI score and the prognostic model were examined by calculating the net reclassification improvement (NRI), as described by Pencina et al. [23].

## Results

Of the 667 patients diagnosed with PE, 57 (8.5%) died within 30 days of hospitalization, and 610 (91.5%) survived at least 30 days after hospital admission (Table 1). All of the patients were followed up 30 days after diagnosis of PE. Causes of death were considered to be related to PE in 33 patients (58%); sepsis (n = 9); cancer (n = 7), major bleeding (n = 4) and other causes (n = 4). PE was diagnosed using spiral chest CT in most patients (n = 635), but was also diagnosed using lung ventilation/perfusion scanning (n = 7), and based on DVT (n = 25). N-terminal prohormone of brain natriuretic peptide (NT pro-BNP) was measured in 322 patients. Complete blood count and serum creatinine levels were measured in 663 patients.

**Table 1:** Clinical characteristics at diagnosis for the 667 patients

		**30-day all-cause mortality**		
**Variable**	**Overall (n = 667)**	**No (n = 610)**	**Yes (n = 57)**	***P *** **value**
Age in years, mean (± SD)	66.4 (± 13.3)	66.9 (± 12.9)	67.8 (± 12.8)	0.60
Male, n (%)	295 (44.2%)	264 (43.2%)	31 (54.3%)	0.27
**Comorbidity, n (%)**				
Heart failure	75 (11.2%)	64 (10.4%)	11 (19.2%)	0.19
Renal dysfunction	96 (14.3%)	79 (12.9%)	17 (29.8%)	0.001
Cancer	181 (27.1%)	161 (26.3%)	20 (35.0%)	0.15
Previous DVT or PE	49 (7.3%)	46 (7.5%)	3 (5.2%)	0.55
Infection	277 (41.5%)	243 (39.8%)	34 (59.6%)	0.006
**Clinical finding, n (%)**				
Hemoptysis	17 (2.5%)	16 (2.6%)	1 (1.7%)	0.91
Chest pain	60 (8.9%)	58 (9.5%)	2 (3.5%)	0.57
Altered mental state*	65 (9.7%)	49 (8.0%)	16 (28.0%)	<0.0001
Systolic blood pressure, mean (± SD), mmHg	121.8 (± 20.6)	123 (± 20.3)	110.5 (± 20.1)	0.0002
Shock^†^	36 (5.3%)	28 (4.5%)	8 (14.0%)	0.0025
Positive spiral CT, n (%)^‡^	635 (95)	583 (95)	52 (91)	–
Main pulmonary artery	247 (37)	220 (36)	27 (47)	–
Lobar artery	235 (35)	218 (35)	17 (30)	0.429
Segmental artery	146 (22)	136 (22)	100 (17)	–
Subsegmental artery	7 (1)	6 (1)	1 (2)	–
**Cardiac chamber ratio and cardiac biomarker, mean**				
RV/LV ratio (± SD) n = 635	1.22 (± 0.45)	1.20 (± 0.43)	1.51 (± 0.55)	0.0006
NT Pro-BNP, ng/L (± SD) n = 322	4906 (± 13381)	3637 (± 13523)	8917 (± 10861)	0.045
**Systemic inflammatory response**				
Heart rate >90 beats/min	187 (28.0%)	157 (25.7%)	30 (52.6%)	0.0003
Respiratory rate >20 breaths/min	248 (37.1%)	212 (34.7%)	36 (63.1%)	0.001
Body temperature (BT) >38.3°C or <36.0°C, n (%)	15 (2.2%)	10 (1.6%)	5 (8.7%)	0.0005
WBC <4,000 or >12,000 mL, n (%)	196 (29.3%)	169 (27.7%)	27 (47.3%)	0.0018
SIRS ≥ 2	191 (28.6%)	157 (25.7%)	34 (59.6%)	<0.0001
SIRS Score (0–4)	0.29 (± 0.45)	0.27 (± 0.44)	0.52 (± 0.50)	<0.0001
SIRS satisfying the WBC criteria, n (%)	113 (16.9%)	90 (14.7%)	23 (40.3%)	<0.0001
**PESI**^**§**^	95.9 (± 29.1)	93.3 (± 26.5)	122.9 (± 39.4)	<0.0001

SD: standard deviation; DVT: deep vein thrombosis; PE: pulmonary embolism; NT pro-BNP: N-terminal prohormone of brain natriuretic peptide; RV: right ventricle; LV: left ventricle; SIRS: systemic inflammatory response syndrome; WBC: white blood cell.

*Altered mental status was defined as disorientation, stupor, or coma.

^†^Shock was defined as systolic blood pressure < 90 mmHg.

^‡^Most proximal anatomic level of pulmonary embolism shown by spiral computed tomography.

^§^PESI: pulmonary embolism severity index [4].

### Prognostic factors

No significant differences in age or sex were observed between the group of patients who died within 30 days of hospital admission and the group of patients who survived. We compared comorbidities identified at the time of PE diagnosis, and found no significant differences between the 2 groups in terms of cardiac failure, active cancer, or history of DVT or PE; renal dysfunction was the only comorbidity in which a significant difference was observed (Table 1).

We compared the clinical symptoms and vital signs of the 2 groups, as recorded at the time of hospitalization. We found no significant inter-group differences in terms of hemoptysis or chest pain; however, pulse rate (98.5 ± 25.2 versus 84.7 ± 14.2, *P* < 0.001), and respiratory rate (25.3 ± 7.4 versus 21.4 ± 4.0, *P* < 0.001) were significantly higher among the group of patients who died within 30 days of admission than that among the group of patients who survived for at least 30 days. Systolic blood pressure was significantly lower in patients who died (110.5 ± 20.1 versus 123 ± 20.3, *P* <0.001) than that among those who survived. Significant inter-group differences were also observed in terms of shock occurrence. N-terminal prohormone of brain natriuretic peptide levels measured at the time of hospital admission, and the RV/LV ratios were significantly higher in the group of patients who died within 30 days.

**Figure 1:**
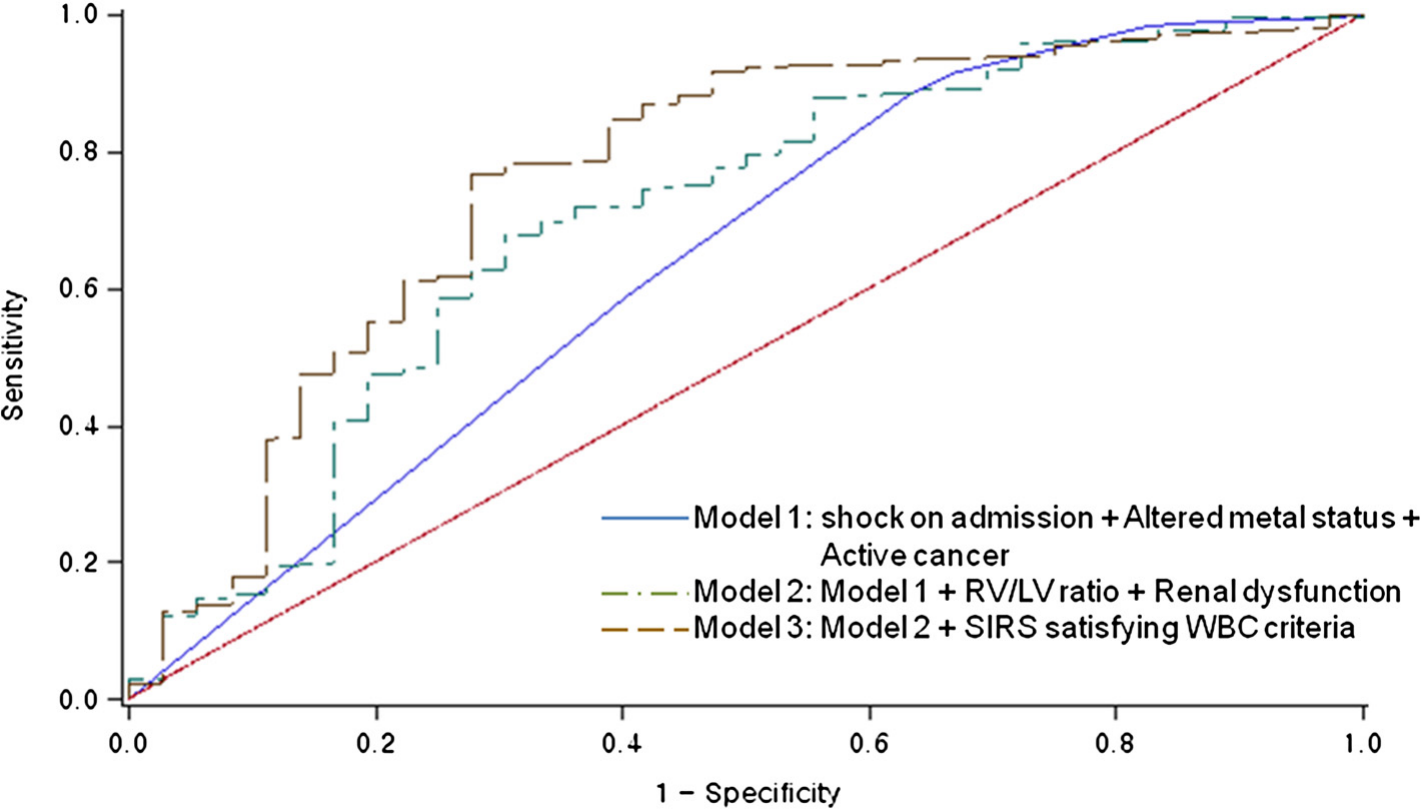
**Receiver operating characteristic curves for the 30-day mortality.** The curves are based on logistic regression models for risk prediction, incorporating the following: Model 1, shock on admission + altered mental state + active cancer (AUC = 0.64; 95% CI, 0.53–0.74); Model 1 + RV/LV ratio + renal dysfunction (AUC = 0.70; 95% CI, 0.59–0.76); Model 1 + RV/LV ratio + renal dysfunction + SIRS satisfying the WBC criterion (AUC = 0.76; 95% CI, 0.66–0.85), *P* = 0.05 versus Model 1. AUC: area under the receiver operating characteristic curve; CI: confidence interval; RV: right ventricle; LV: left ventricle; SIRS: systemic inflammatory response syndrome; WBC: white blood cell.

All SIRS criteria were significantly less common within the survivor group (Table 1). Multivariate analysis showed that all 4 SIRS criteria were significantly associated with mortality (Table 2). Univariate analysis of SIRS satisfying the WBC criterion, RV/LV ratio, altered mental state, renal dysfunction, infection, and shock identified all of these variables as statistically significant. However, according to multivariate analysis, altered mental state, shock, RV/LV ratio, and SIRS satisfying the WBC criterion were statistically significant, while infection and renal dysfunction were not (Table 3). Based on these variables, the AUC was calculated at 0.74 (95% CI, 0.64–0.83) (Figure 1).

**Table 2:** Univariate and multivariate analyses of SIRS criteria

	**Univariate analysis**		**Multivariate analysis**	
**Variable**	**OR (95% CI)**	***P *** **value**	**OR (95% CI)**	***P *** **value**
Body temperature >38.5°C or <36.0°C	5.7 (1.8–17.4)	0.002	4.6 (1.4–14.8)	0.008
Heart rate > 90 beats/min	3.2 (1.8–5.5)	<0.001	2.0 (1.1–3.6)	0.019
Respiratory rate > 20 breaths/min	3.2 (1.8–5.6)	<0.001	2.5 (1.4–4.6)	0.022
WBC <4,000 or >12,000 mL	2.3 (1.3–4.0)	0.023	1.9 (1.1–3.5)	0.018

SIRS: systemic inflammatory response syndrome; OR: odds ratio; bpm: beats per minute; WBC: white blood cell.

**Table 3:** Risk factors for 30-day mortality according to multivariate analysis, model 1

	**Complete case analysis (n = 635)**	
**Variable**	**OR (95% CI)**	***P *** **value**
**Altered mental state**^**†**^	4.0 (1.8–8.7)	< 0.001
**Infection**	1.5 (0.8–2.9)	0.15
**Cancer**	1.7 (0.7-5.1)	0.18
**Shock**	2.6 (1.0–7.1)	0.04
**Renal dysfunction**	1.8 (0.8–4.0)	0.09
**RV/LV ratio**	1.7 (1.0–2.8)	0.04
**SIRS satisfying WBC criterion**	2.8 (1.5–5.4)	0.001

OR: odds ratio; RV: right ventricle; LV: left ventricle; SIRS: systemic inflammatory response syndrome; WBC: white blood cell.

^†^Altered mental status was defined as disorientation, stupor, or coma.

### Systemic inflammatory response satisfying the WBC criteria as a prognostic factor of 30-day mortality of PE patients

Three multivariate logistic regression models were developed: the first model was a clinical model that included clinical variables; the second model was based on the RV/LV ratio and laboratory variables; the final model was based on SIRS (Table 2). In each model, altered mental state, shock, active cancer, RV/LV ratio, and all SIRS criteria were independently associated with 30-day mortality. Furthermore, the association was statistically significant for the RV/LV ratio and all the SIRS criteria. According to the final multivariate model, altered mental state, RV/LV ratio, and SIRS satisfying the WBC criterion were independently associated with the 30-day mortality of the 635 patients whose data were complete (Table 3). The variables included in the model accounted for 7.0% of the variation in individual 30-day mortality. The final model had a strong predictive performance, with an AUC of 0.76 (95% CI, 0.66–0.85) for all 635 patients (Figure 1). However, no statistically significant difference was observed between the ROC curves of the different models. The internal validity of the predictive model was checked by bootstrapping.

Patients were reclassified according to 30-day mortality based on the original clinical model and final model adding RV/LV Ratio, renal dysfunction, SIRS satisfying the WBC criterion (Table 4). For the 30-day non-survivors, classification according to the final model was more accurate for 20 patients and was not less accurate for any patients, compared to classification according to the clinical model. For the 30-day survivors, classification according to the final model was more accurate for 17 patients and less accurate for 84 patients, compared to classification according to the clinical model. The NRI achieved by the final model was estimated at 24.0% (*P* < 0.001).

**Table 4:** Reclassification of patients according to 30-day mortality based on the clinical model, RV/LV ratio, renal dysfunction, and SIRS satisfying the WBC criterion (Complete Case Analysis, n = 635)

**Clinical model: shock on admission +altered mental state + active cancer**	**Clinical model + RV/LV ratio + renal dysfunction + SIRS satisfying the WBC criterion**			
	**<10%**	**10%–30%**	**≥30%**	**Total**
Non-survivors				
<10%	20	**15**	**0**	35
10%–30%	0	11	**5**	16
≥30%	0	0	5	5
Total	20	26	10	56
Survivors				
<10%	438	**72**	**3**	513
10%–30%	*17*	35	**9**	61
≥30%	*0*	*0*	5	5
Total	455	107	17	579

PE: pulmonary embolism; RV: right ventricle; LV: left ventricle; SIRS: systemic inflammatory response syndrome; WBC: white blood cell.

Reclassification tables for survivors or non-survivors of PE were constructed using PE risk categories [23], based on mortality prediction obtained by the clinical model and the clinical model + RV/LV ratio + renal dysfunction + SIRS satisfying WBC criterion, and included 3 categories: <10%, 10%–30%, and ≥30% of 30-day mortality.

For the 56 non-survivors, classification improved using the model with RV/LV ratio + renal dysfunction + SIRS satisfying the WBC criterion, but worsened for 20 subjects (cells in *bold*). For the 579 survivors, 17 subjects (cells in *italics*) were reclassified down and 84 subjects (cells in *bold*) were reclassified up.

### Leukocytes as a prognostic factor of 30-day mortality after PE

**Figure 2:**
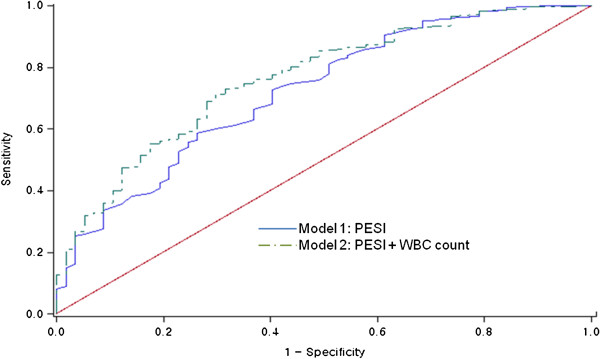
**Receiver operating characteristic curves for the 30-day mortality.** The curves are based on logistic regression models for risk prediction, incorporating the following: Model 1, PESI (AUC = 0.72; 95% CI, 0.68–0.75); PESI + WBC count (AUC = 0.76; 95% CI, 0.72–0.79), *P* = 0.008 versus Model 1. PESI: pulmonary embolism severity index; AUC: area under the receiver operating characteristic curve; CI: confidence interval; WBC: white blood cell.

Both PESI scores and WBC counts were independently associated with the 30-day mortality rate (Table 5). The variables included in the model accounted for 11.8% of the variation in individual 30-day mortality. The final model had a strong predictive performance, with an AUC of 0.76 (95% CI, 0.72–0.79) for all 654 patients studied (Figure 2). There was a statistically significant difference (*P* < 0.001) between the ROC curves of the PESI and the PESI + WBC count models. The internal validity of the predictive model was checked by bootstrapping.

**Table 5:** Risk factors for 30-day mortality according to multivariate analysis, model 2

	**Complete case analysis (n = 654)**	
**Variable**	**OR (95% CI)**	***P *** **value**
**PESI**	1.03 (1.02–1.38)	<0.01
**WBC**	1.05 (1.01–1.09)	0.01

PESI: pulmonary embolism severity index; OR: odds ratio; WBC: white blood cell.

Patients were reclassified according to 30-day mortality (Table 6); for the 30-day non-survivors, classification according to the PESI + WBC count model was more accurate for 4 patients and less accurate for 1 patient, compared to classification according to PESI alone. For the 30-day survivors, classification according to the final model was more accurate for 35 patients and less accurate for 18 patients, compared to classification according to the clinical model. The NRI achieved by the final model was estimated at 8.1% (*P* = 0.04). Thrombolytic drugs, inferior vena cava filter, and surgical embolectomy were used in 16, 7, and 2 of the cases, respectively.

**Table 6:** Reclassification of 30-day survivors and non-survivors based on PESI + WBC count (Complete Case Analysis, n = 654)

**PESI**	**PESI + WBC count**			
	**<10%**	**10%–30%**	**≥30%**	**Total**
Non-survivors				
<10%	26	**3**	**0**	29
10%–30%	*0*	15	**1**	16
≥30%	*0*	*1*	11	12
Total	26	19	12	57
Survivors				
<10%	453	**13**	**1**	467
10%–30%	*34*	79	**4**	117
≥30%	*0*	*1*	12	13
Total	487	93	17	597

PESI: pulmonary embolism severity index; WBC: white blood cell.

Reclassification tables for survivors or non-survivors of PE were constructed using PE risk categories [23], based on mortality prediction obtained by PESI + WBC count. For the 57 non-survivors, classification improved using the model with PESI + WBC count; 1 subject (cell in *underline*) was reclassified down and 4 subjects (cells in *bold*) were reclassified up. For the 579 survivors, 35 subjects (cells in *italics*) were reclassified down and 18 subjects (cells in *bold*) were reclassified up.

## Discussion

The independent variables identified in this study as predictors of mortality within 30 days of hospital admission for PE were: SIRS satisfying the peripheral blood WBC count criterion, altered mental state, shock, and the RV/LV ratio. In patients with PE, WBC count and SIRS satisfying the peripheral WBC count criteria were significantly associated with mortality within 30 days of hospital admission (OR = 1.05, 95% CI, 1.01–1.09; OR = 2.8, 95% CI, 1.5–5.4, respectively) (Tables 2, 3).

Patients with PE initially present with various clinical profiles, ranging from clinically stable to shock status. Moreover, 30-day mortality rates in PE patients with hemodynamic abnormalities have been reported to range from 5% to 58% [1,24]. The prevalence of shock in our sample was 5.3% (36 cases). The 30-day mortality rate for PE patients presenting with shock was 25%. The RV/LV ratio measured by chest CT indicated potential right ventricular failure, which also reflects the severity of PE [20]. The mean RV/LV ratio observed in the present study was similar to that reported in a previous study [19]. Moreover, the RV/LV ratio was significantly associated with 30-day mortality (OR = 1.7, 95% CI, 1.0–2.8), which is consistent with previous reports that death within 30 days of hospital admission is predictable [25]. However, the levels of N-terminal prohormone of brain natriuretic peptide were not independently associated with mortality our study. This may be, in part, due to the limitations imposed on data collection, which are inherent to retrospective studies.

The percentage of PE patients with 3 or more risk factors, including infection, has been reported to be as high as 50% [26]. This is expected, as WBCs are involved in the coagulation process [27]. In the present study, we demonstrated that in addition its role as a risk factor for PE, SIRS also plays an important role in patient prognosis. Analysis of SIRS criteria showed that heart rate and the WBC criterion were significantly associated with mortality (Table 2). Multivariate analysis including all significant variables, identified SIRS satisfying the WBC count criterion as a significant prognostic factor (Table 3). These results indicate that WBC count in the systemic inflammatory response is an important prognostic factor in PE patients. Although there was a significantly higher percentage of infection in non-survivors compared to survivors (Table 1), multivariate analysis showed SIRS satisfying leukocytes criteria was an independent predictor of outcome, while infection was not. These results suggest that SIRS satisfying the leukocytes criteria is a predictor of outcome, independent of coincident events such as an infection.

In contrast to studies that reported a significant association between the presence of a malignant tumor and PE mortality [1,28], no such relationship was observed in this study. We speculate that this lack of association may be due to the recent progress in cancer management. Indeed, this may also result in a difference in the ROC curve from the original PESI [4]. Cancer survival rates are currently improving [29-31], which may influence the reclassification of the prognostic index in the future.

The area under the ROC curve of shock + altered mental state + active cancer was 0.64, which is lower than the previously reported value [28]. However, when RV/LV ratio, renal dysfunction, and SIRS satisfying the WBC criterion were included in the risk prediction model, a marginal increase in the AUC from 0.64 to 0.76 (*P* = 0.05) was observed. We classified the predicted risks obtained from both models (old and new) into 3 categories (0%–10%, 10%–30%, >30% of 30-day PE mortality) and then cross-tabulated these 2 classifications. Consequently, classification was less accurate for approximately 36% of non-survivors according to the new model, compared to the old model. In contrast, classification was more accurate for approximately 11% of survivors (Table 4).

The prognostic significance of WBC count was evaluated using the previously established PE prognostic index. Huang CM et al. also showed WBC count (≥11,000 mm^3^) was an independent predictor of 30-day mortality in PE patients [32]. Accordingly, we found that WBC count was an independent prognostic factor, apart from PESI, for 30-day PE mortality (Table 5). The area under the ROC curve of PESI was 0.68, which is lower than the previously reported value [4]. However, when WBC count was added to the PESI, an increase in AUC from 0.72 to 0.76 (*P* = 0.008) was observed. We classified the predicted risks obtained from both models (PESI and PESI + WBC) into 3 categories (0%–10%, 10%–30%, >30% of 30-day PE mortality), and then cross-tabulated these classifications. Consequently, the classification of approximately 5% of non-survivors was less accurate when comparing PESI + WBC with PESI alone. Conversely, approximately 3% of survivors were reclassified down when WBC count was added to PESI (Table 6).

Thrombolytics, inferior vena cava filter, and surgical embolectomy were performed in 16, 7, and 2 of the cases included in this study, respectively. Thus, our analysis of prognostic factors did not account for treatment method.

## Conclusions

The independent prediction factors that demonstrated a significant correlation with mortality within 30 days of hospital admission were altered mental state at the time of hospital admission, RV/LV ratio, SIRS satisfying the WBC criterion, and WBC count. We suggest that careful assessment of systemic inflammatory responses and WBC count is necessary in determining the prognosis of PE patients.

## Competing interests

All authors declare that they have no competing interests.

## Authors’ contributions

JYJ was responsible for data analysis, and for drafted this manuscript. WIC was responsible for the content of the manuscript, for study design, for data analysis, and for drafted this manuscript; JWL was responsible for the data collection; BHR was responsible for the data collection and analysis; MYL was responsible for the data analysis and interpretation. All authors contributed to the drafting and revisions of the manuscript. All authors read and approved the final manuscript.

## Pre-publication history

The pre-publication history for this paper can be accessed here:

http://www.biomedcentral.com/1471-2466/13/74/prepub
